# Assessing the adoption and utilization of the Rwanda COVID-19 data analytics system: a mixed methods approach

**DOI:** 10.1093/oodh/oqae034

**Published:** 2024-08-28

**Authors:** Hassan Mugabo, Gilbert Rukundo, Jean Claude S Ngabonziza, Jean-Baptiste Mazarati, Joseph Aghatise, Olukunle Akinwusi

**Affiliations:** Research Innovation and Data Science, Rwanda Biomedical Center, Kigali, Rwanda (RBC), KG 644 St, Kigali, Kimihuruna, P.O. Box 7162, Kigali, Rwanda; Research Innovation and Data Science, Rwanda Biomedical Center, Kigali, Rwanda (RBC), KG 644 St, Kigali, Kimihuruna, P.O. Box 7162, Kigali, Rwanda; Research Innovation and Data Science, Rwanda Biomedical Center, Kigali, Rwanda (RBC), KG 644 St, Kigali, Kimihuruna, P.O. Box 7162, Kigali, Rwanda; Digital Health, Foundation for Innovative New Diagnostics, Geneva, Switzerland Chemin du Pommier 40 1218 Grand-Saconnex, Switzerland; Digital Health, Foundation for Innovative New Diagnostics, Geneva, Switzerland Chemin du Pommier 40 1218 Grand-Saconnex, Switzerland; Digital Health, Foundation for Innovative New Diagnostics, Geneva, Switzerland Chemin du Pommier 40 1218 Grand-Saconnex, Switzerland

**Keywords:** digital health, disease surveillance, impact assessment, usability, interoperability

## Abstract

Introduction: Rwanda has been widely lauded for its exceptional response to the COVID-19 pandemic. However, although Rwanda established a national system for COVID-19 testing and vaccination data, concerns have been raised about data fragmentation which requires linkage of various data sources, access to data for real-time decision-making, and data completeness. Methods: We assessed the adoption of the Rwanda COVID-19 data Analytics System (RCAS) for public health staff that employ data from various platforms to generate evidence for policy- and decision-making. A random sample of 56 participants was drawn from the 98 who attended the 2022 RCAS training for data managers from the Rwanda Biomedical Center, technical partners, and health facilities. Of the selected participants, 42 completed the online self-administered questionnaire within the 14-day data collection period. Key informant interviews were then conducted with a subset of 14 respondents. Results: A strong positive relationship (χ^2^ = 9.1049, *P* < 0.05) emerged between respondents' decision-making regarding RCAS and their support for its sustainability. There was a marginal association (χ^2^ = 3.3358, *P* = 0.059) suggesting a link between users' ease of data exchange through RCAS and their support for its long-term sustainability, warranting further exploration. Conclusion: RCAS had a positive impact on improvements in data linkage, access to individual-level data for analyses, and progress toward harmonization of health data beyond COVID-19 in Rwanda. Users noted the usability, acceptability, and interoperability of the system. Recommendations for further improvement and scaling of the intervention are discussed.

## INTRODUCTION

The global COVID-19 pandemic challenged healthcare systems worldwide, demanding swift and innovative responses. This was particularly true for low- and middle-income countries (LMICs) already grappling with limited resources and complex healthcare disparities. In the wake of the novel coronavirus 2019 (COVID-19) pandemic, LMICs rapidly developed prevention and response measures aimed at stopping the spread of the disease. Notable among these measures were ‘lockdowns’, to reduce physical contact between individuals [1], and the large-scale rollout of digital technologies to help identify and report COVID-19 cases. Rwanda is a LMIC in East Central Africa, with an estimated population of ~13 246 394 people [2]. In Rwanda, testing and linkage to care (a test, trace and isolate (TTI) strategy) was one of the key strategies implemented to reduce the spread of COVID-19.

This approach hinged on extensive COVID-19 testing, aiming to quickly identify infected individuals and contain transmission. Rwanda's ambitious testing campaign targeted nearly half of its population, conducting >5.8 million tests throughout the pandemic, translating to around 6207 daily tests [3]. This impressive testing rate, surpassing many high-income countries, yielded >132 500 confirmed cases, indicating a positivity rate of 2.27% [3]. Rwanda's ambitious vision for data-driven public health has driven significant investments in infrastructure and systems. One such investment involved the development of a central data warehouse within the Ministry of Health [4]. This warehouse initially focused on disease-specific data collection and storage using DHIS2, a widely adopted health information system [5]. While this approach proved effective in its early stages, the increasing volume of health data from diverse sources and growing demand for holistic analysis necessitated a new approach.

The burgeoning number of concurrent data transactions on the central server began to pose limitations. Frequent requests from analysts seeking comprehensive insights across the entire healthcare system highlighted the need for a more integrated system capable of handling concurrent access and facilitating holistic analysis. This development signifies a crucial turning point in Rwanda's data-driven health strategy, moving from isolated disease-specific data to a more interconnected and holistic approach. Understanding the factors driving this shift and exploring the challenges and opportunities presented by integrating diverse healthcare data sets are crucial steps in evaluating the future of Rwanda's health information infrastructure.

The Rwandan COVID-19 Analytics System (RCAS) sits at the heart of the country's data-driven approach to managing the pandemic. Mirroring the central DHIS2 server for COVID-19 testing and vaccination databases, RCAS offers a crucial bridge between raw data and actionable insights. It serves as a centralized platform for data interoperability, facilitating access and analysis of critical COVID-19 information for researchers, policymakers and healthcare professionals.

Beyond simply storing data, RCAS acts as a vital communication hub, enabling seamless interaction with other digital systems within the Rwandan healthcare ecosystem. This interoperability layer breaks down data silos, ensuring holistic analysis of COVID-19 trends across various platforms. By providing unified access to the vast repository of testing and vaccination data, RCAS empowers stakeholders to perform nuanced analyses, track vaccine effectiveness, and make informed decisions based on real-time insights.

The foundation of RCAS lies in the concept of data replication, a well-established technique for enhancing database performance and availability [6]. By replicating the central DHIS2 databases, RCAS creates a readily accessible version optimized for analytical tasks. This approach offers several advantages:

- Improved query performance: Analysts can access and interrogate COVID-19 data on a dedicated platform designed for high-volume analytical workloads, minimizing impact on the operational DHIS2 system.
- Enhanced disaster recovery: Replication safeguards against potential data loss on the central DHIS2 COVID-19 server, ensuring continuous access to critical information even in unforeseen circumstances.
- Scalability and flexibility: The flexibility of the RCAS architecture allows for seamless integration of new data sources and analytical tools as needed, future-proofing the platform for evolving public health needs.

RCAS represents a vital cog in Rwanda's comprehensive COVID-19 response strategy. Its dedication to data interoperability, accessibility, and performance underpins the country's ability to make informed decisions, optimize resource allocation, and ultimately save lives.

### Pre-RCAS dataflow model

In the era preceding the RCAS, Rwanda's healthcare data landscape was characterized by fragmentation and hindered accessibility Data resided in isolated silos, scattered across multiple disparate databases, each with its own distinct format and access protocols. This fragmented infrastructure posed significant challenges to researchers and analysts seeking to harness the power of this data for critical insights and informed decision-making [7].

**Figure 1:**
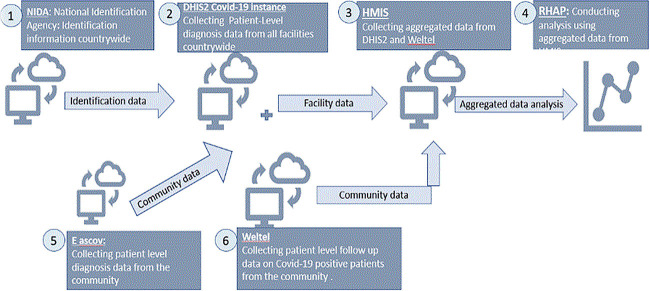
Overview of pre-RCAS dataflow

The process of accessing and integrating data from these disparate sources was often cumbersome and time-consuming. Valuable time and resources were frequently expended in navigating compatibility issues, deciphering inconsistent data structures and bridging communication gaps between different data custodians. During project landscaping, fragmentation of data was a key discovery. There was no link between COVID-19 testing data, contact tracing data and vaccination data as shown in Fig. 1. In [8] study, emphasizes was on the need for a holistic approach, advocating for unified data platforms, standardized data collection practices, and capacity building for data management and analysis. The study underscores the important of avoiding data fragmentation when planning for diseases response. The DHIS2 production server for the COVID-19 data system experienced performance degradation and increased downtime as user concurrency rose [9]. This was compounded by the system's concurrent use for data entry, analysis, and third-party access.

### Objectives

1. To assess the adoption, interoperability, reporting, and functionality of RCAS in Rwanda.
2. To assess the utilization of data generated from RCAS for the COVID-19 response in Rwanda.
3. To identify opportunities for the expansion and scale-up of RCAS.

The assessment team focused on the following five core domains of digital health systems: Leadership and governance, ICT infrastructure, management and workforce, data quality, and interoperability.

## METHODS

### Study design

The study included 56 participants drawn randomly from a list of 98 data managers, RBC staff, and RBC partners who participated in RCAS training facilitated by RBC in 2022. The RCAS assessment questionnaire was developed in three phases. The first phase involved the design of the conceptual framework for the questionnaire. A systematic literature review was conducted to explore various frameworks that can be used for digital health project assessment. The second phase involved converting the assessment questionnaire to a digital format using Rwanda’s REDCap tool that was approved for research data capture at the time of the assessment. In the third phase, the digital questionnaire was pre-tested for integrity and ease of use. The questionnaire was then modified to address any gaps identified and improve its administration. A digital health project maturity model [10] was used for the assessment, with a mixed methods approach for data collection. A maturity model describes the process components in any project that can be adjusted to lead to improved outputs and outcomes; in the present case, outcomes can be classified as any of the following:

- Level 1: Initial - RBC has implemented RCAS, but its usage is sporadic and inconsistent.
- Level 2: Managed - RBC has defined processes for using RCAS, but they are not followed consistently.
- Level 3: Integrated - RCAS has been integrated into RBC’s workflows and processes, and its usage is consistent across the departments where it has been deployed.
- Level 4: Optimized - RBC has optimized the use of RCAS to improve data-driven decision-making processes.

### Inclusion criteria

To be eligible for inclusion in the study, participants had to meet one of the following criteria:

- Health facility COVID-19 data managers who attended RCAS training in 2022.
- RBC technology partners who have established integration with the RCAS platform.
- Data Science staff who worked at RBC central office that used RCAS for periodic reporting, research, surveillance, or other purposes.

### Data collection

Primary data for this study were collected via a questionnaire. This structured questionnaire, comprising both open and closed questions, was developed and digitized using the REDCap platform, before being distributed online to collect data from the respondents. The questionnaire was circulated to the study participants who had been identified (n = 56), for self-administration. The assessment was conducted based on questionnaires completed by respondents and returned within 14 days (n = 42). One-on-one key informant interviews were conducted with 14 randomly selected respondents to obtain additional qualitative data to help inform the analysis of the quantitative data obtained from the questionnaires.

### Data analysis

The data collected were analyzed by descriptive statistics, including frequencies and percentages, and the chi-square test to investigate the relationships between some variables. Content analysis was used to analyze the responses to open-ended questions on the questionnaire, which provided additional information to that obtained from the closed questions.

### Response rate

The response rate was calculated and acceptance of responses was based on two criteria: (i) Consent to participate in the assessment and (ii) submission of a completed questionnaire within the 14-day period of the assessment. A total of 42 (75%) respondents completed and submitted their self-administered questionnaire within the allocated period.

### Overview of the RCAS platform

**Figure 2:**
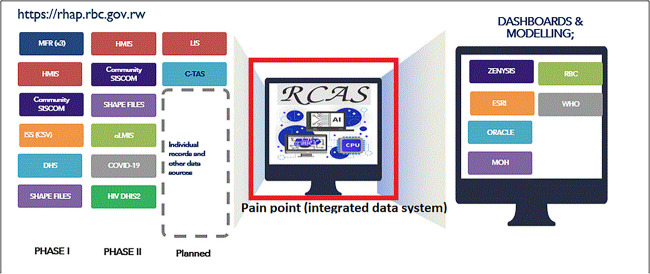
RBC pain point addressed by RCAS platform (source: RBC data science 2023)

RCAS is a web-based platform. Within a remarkable 9-month period in 2022, RCAS sprang to life. A thorough landscape analysis, informed by major stakeholders, revealed critical gaps in Rwanda’s COVID-19 data capture, reporting, and research. RCAS tackled these head-on, as showcased in Fig. 2. Today, RCAS transcends its initial purpose, enabling data integration from diverse sources and diseases, empowering informed responses and decision-making for a future beyond COVID-19.

**Figure 3:**
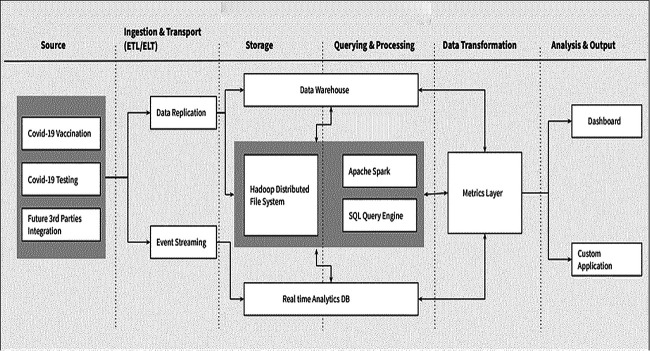
RCAS system architecture

Figure 3 shows the RCAS architecture and dataflow layers for integration with other digital health systems used for COVID-19 data management. RCAS was conceived and implemented to bridge the gap between COVID-19 data capture and reporting systems while ensuring expanded access to individual-level data for real-time responses to outbreaks of disease, without interrupting the production server. RCAS provides end-users with an interface to access the COVID-19 data held in the system by offering a mirrored version of the COVID-19 DHIS2 server for testing and vaccination databases. This enables data analysis to be carried out, the results of which can be used to provide detailed evidence to decision-makers and help guide surveillance initiatives in the case of similar outbreaks. Integration of the system with existing data systems also allows for the extraction of data from other disease programs, allowing visibility of patterns across the various disease programs*.*

### RCAS data sources

The source databases for RCAS are DHIS2 instances (testing and vaccination), with integrated community tracking data. Periodic mirrors of the DHIS2 productive server databases are synchronized with the RCAS platform through an automated system for data sharing. Mirrors of production server databases can provide uninterrupted, real-time access to data for decision making. End-users who have access to the RCAS platform can write queries that range from simple to complex, to generate custom reports and dashboards for their specific needs. This includes the ability to integrate with third-party systems, such as the Rwanda Health Analytics Platform (RHAP) and other digital systems, through application program interface (API) calls based on representational state transfer (REST).

### Training in RCAS and its implementation

**Figure 4:**
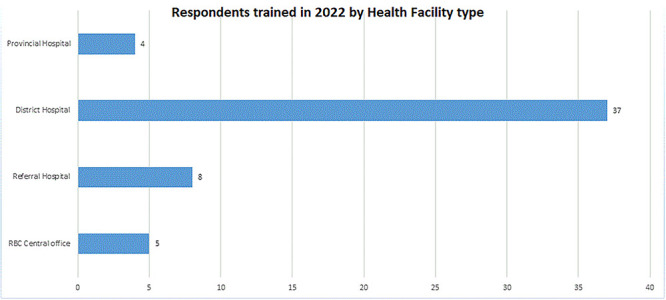
Respondents trained in 2022, by health facility type

Training of trainers was conducted for members of the RBC data science team, and these trainers then facilitated end-user training. A total of 54 data managers (Fig. 4) from various healthcare disciplines, participated in the training, which lasted for 10 days in December 2022.

## RESULTS

### Demographic information

#### Gender distribution of the respondents

The survey garnered a strong response, with 79% of Respondents identifying as male and 21% as female. Although the survey achieved its target response rate of 72%, the notable gender gap (79% male, 21% female) highlights the need for future research to ensure inclusivity.

Distribution of respondents based on whether they were trained in the use of RCAS.

Encouragingly, a substantial majority (64.29%) of respondents, 27 participants in total, received RCAS training. This suggests a strong understanding of the platform and its potential impact on daily work and individual performance. Subsequent questions probed deeper into their RCAS-based knowledge and data access needs for informed decision-making.

#### Distribution of respondents based on their work location

A clear majority (80.95%) of respondents were data managers at district hospitals, with only 4.76% working at the RBC central office. District hospitals were the primary workplace for data managers, with 80.95% of respondents coming from this setting, compared to 4.76% at the RBC central office.

Distribution of the respondents according to the departments/units where they worked

The high representation of respondents from data management (59.52%) and monitoring and evaluation (28.57%) units, totaling over 88%, underscores the significance of data in their daily activities (Table 1).

**Table 1:** Units/departments of respondents

Unit/Department	Frequency	Percentage
Statistics and epidemiology	2	4.76
Monitoring and evaluation	12	28.57
Research and planning	1	2.38
Facility data management	25	59.52
RBC partner	2	4.76
Total	42	100

Data usage takes center stage, as Table 1 shows: 59.52% of respondents work in data management and 28.57% in monitoring and evaluation, emphasizing the reliance on data-driven insights in these roles.

### Objective 1: Assessment of adoption, interoperability, reporting, and functionality of RCAS in Rwanda

Weik et al investigated the factors influencing the adoption of digital health systems among healthcare workers in a developing economy. Their findings emphasize the significance of perceived usefulness [11], ease of use and adequate training in driving successful adoption rates [12].

#### Adoption of RCAS

The results shown in Table 2a indicate that > 75% of respondents attested to the availability of the internet. Reliable internet connectivity significantly facilitated access to the RCAS system, leading to a higher adoption rate among stakeholders. In terms of downtime experienced, 71.43% of respondents reported that they had not experienced any downtime (Table 3). The high percentages recorded for internet availability and the absence of downtime suggest that the RCAS system efficiently provided readily available data as and when data were needed for decision making. However, some staff opined that there were issues related to poor data quality and suggested ways to improve this. The measures they suggested included the regular use of RCAS to help detect quality issues, refresher training for users of RCAS, periodic updates of data held on RCAS (daily or weekly), and the provision of periodic feedback. During the key informant interviews, it was discovered that one of the key benefits of the RCAS platform was in helping to identify COVID-19 data issues. In terms of standards and interoperability, RCAS has been optimized (90.5% digital maturity score). This outcome could be attributed to the flexibility of data exchange and the availability of local support within RBC.

**Table 2:** Key requirement for RCAS functionality

Reference	Question	Strongly agree	Agree	Disagree	Strongly disagree	Not aware	Total
2(a)	Availability of internet connectivity	5	27	7	0	3	42
2(b)	Availability of ICT support	6	24	3	7	2	42
2(c)	Proficiency of local ICT support to address any RCAS problems	3	28	4	3	4	42
2(d)	Availability of programs for training on the use of RCAS	3	26	5	1	7	42
2(e)	Human resources in place to provide continuous training for users of RCAS	6	23	7	0	6	42

**Table 3:** RCAS server downtime

Response	Frequency	Percentage
No	30	71.43
Yes	12	28.57
Total	65	100

#### Interoperability of RCAS with other digital systems

In terms of the interoperability of the system, 71.43% of respondents confirmed they were very familiar with digital interoperability (Table 4), while 59.53% of respondents reported that the exchange of COVID-19 data between them being captured by DHIS2 and transferred to RCAS occurred within one week. The majority of respondents (59.52%) were satisfied or highly satisfied with the interoperability of the system. However, nine staff (21.43%) were not satisfied with the interoperability of the system and suggested some improvements, which included expanding RCAS functionality to include other diseases and more training and re-training of RCAS users. Qualitative data collected during the key informant interviews suggested priority diseases for the expansion of RCAS should include hepatitis, HIV, malaria, tuberculosis (TB), and possibly other diseases.

**Table 4:** Familiarity with digital interoperability

Response	Frequency	Percentage
Strongly agree	30	71.43
Agree	12	28.57
Total	42	100

#### Functionality of RCAS

Table 2(b) shows that 30 of the 42 respondents attested to the fact that there was local ICT support available to help operate RCAS. It was also reported by 73.81% of respondents (Table 2(c)) that the local ICT support was proficient in addressing any problems related to the use of the RCAS platform. This minimizes technical barriers and ensures smooth system operation. Tables 2(d) and 2(e) show that approximately 69% of the respondents stated that there were programs and human resources available to provide training and re-training services for RCAS users. These findings imply that RBC has the necessary tools and personnel to enable RCAS to function effectively.

### **Objective 2:** To assess the utilization of data generated from RCAS for the COVID-19 response in Rwanda

A resounding 77.5% of respondents (Table 5) agreed that they could tailor RCAS to track the specific COVID-19 indicators most relevant to their needs, empowering them to generate detailed and versatile custom reports. Customization in this context refers to tailoring or adapting RCAS to meet specific requirements or needs. This finding indicates that the RCAS has been appropriately designed to track and analyze key indicators that are relevant and specific to the Rwanda’s COVID-19 response. The platform has features that enable individual-level customization of reports and indicators through a query interface and a customizable dashboard, allowing more informed decision-making, resource allocation, and public health interventions. The option to create customized reports enhances user satisfaction and adoption of digital health systems, as users can access the information they need in a format that suits their preferences [13]. These findings also align with Xiang’s suggestions for ensuring compliance with data privacy regulations through customized reporting in digital health systems [14].

**Table 5:** National and sub-national minimum core indicators are customizable in RCAS

Response	Frequency	Percentage
Strongly agree	12	30.00
Agree	19	47.50
Disagree	2	5.00
Strongly disagree	1	2.50
Not aware	6	15.00
Total	40	100

Analysis revealed that RCAS data has become a central driver of decision-making for the vast majority (73.81%) of users, empowering them to allocate resources, formulate policies, and implement programs with greater accuracy and efficiency. This finding suggests that a majority of respondents utilized data from the RCAS to inform their decision-making processes related to the COVID-19 pandemic. Thus, RCAS is a valuable tool for decision-makers in Rwanda, which enabled them to make informed and evidence-based choices in managing the pandemic and implementing public health interventions. Crucially, leadership and governance structures are in place to sustain RCAS beyond the pilot phase.

### Objective 3: Opportunities for the expansion and scale-up of RCAS

Table 6 shows that ~93% of respondents felt that RCAS should be sustained. This may not be unconnected with its importance in accelerating reporting and decision making. The chi-square test was used to determine whether there was a significant relationship between decision-making and whether RCAS should be sustained**.** The results indicated that a statistically significant relationship (χ^2^ = 9.1049, *P* = 0.011) existed between decision making and support for sustaining RCAS. It was also established that there was a statistically significant relationship (χ^2^ = 3.3358, *P* = 0.059) between satisfaction with the level of interoperability of RCAS and support provided for its sustainability. This may not be unconnected with the added value RCAS provides and which allows for interoperability with other digital health systems. Some of the opportunities for sustainability as reported by respondents included the potential to aggregate data from diverse sources, which is invaluable for research and population health analysis.

**Table 6:** The use of RCAS should be sustained

Response	Frequency	Percentage
No	3	7.14
Yes	39	92.86
Total	42	100

### Key informant interview

In a follow-up phase to the administration of questionnaires to data managers across health facilities and other users of RCAS, in-depth KIIs involving open-ended questions were conducted to obtain more insights and nuances about the suitability and usability of RCAS platform. Zenysis, through its Rwanda Health Analytical Platform (RHAP), is a frequent user of RCAS. Its technology integrates siloed data from various disease information systems, e.g. for COVID-19, HIV and malaria, into their analytical platform to produce dashboards and structured information for the MoH, RBC and health facilities to use. The RBC, at a central level, recognized that RCAS was above all a necessity for their current overall digital architectural platforms. It was the missing link, the solution to the data challenge faced by RBC.

## DISCUSSION

Our evaluation of RCAS revealed several important insights relating to its impact and effectiveness in managing COVID-19 data and supporting decision-making in Rwanda’s pandemic response. Here, we summarize the strengths of RCAS, the challenges of its implementation, and its implications for public health. Our initial findings about the efficiency and availability of RCAS showed >75% of respondents attested to the availability of internet access, indicating that RCAS was well-supported by a reliable internet connection, which is crucial for its effective functioning. Additionally, the limited downtime reported by 71.43% of respondents reflects the system’s reliability and availability, ensuring readily accessible data for decision-making processes. These efficiency metrics suggest that RCAS was successful in providing timely and reliable data to aid COVID-19 response efforts.

Another finding was that there were staff concerns around poor data quality; suggestions to improve this included regular usage of RCAS, refresher training, and regular data updates and provision of feedback. These findings highlight the importance of ongoing data quality management for digital health systems such as RCAS. Although the system may have been efficient in its data provision function, addressing any data quality issues is vital for maintaining the accuracy and integrity of the information used for decision-making.

The key informant interviews revealed that RCAS played an important role in identifying COVID-19 data issues, emphasizing its value as surveillance and monitoring tool. Moreover, the system’s high score for digital maturity, in terms of its standards and interoperability (90.5%), underlines its ability to exchange data effectively and work seamlessly within Rwanda’s health system. This interoperability has contributed to the effectiveness of RCAS as a comprehensive health information platform.

Integrated healthcare allows providers to address multiple interconnected health conditions simultaneously, offering more holistic and comprehensive care. This is crucial for patients with complex needs, where conditions are often interrelated, such as diabetes and depression, or heart disease and hypertension. By treating conditions together, healthcare systems can reduce complications, improve overall outcomes, and simplify the care experience for patients [15]. Digital health projects can contribute to the early detection of disease outbreaks and real-time disease surveillance when expanded to cover different diseases. The timely identification of outbreaks and monitoring of disease trends are critical for effective public health interventions [16]. RCAS has demonstrated efficiency in the management of COVID-19 data, and feedback from the interviews suggests the possibility of expanding the system to include other diseases of interest. Integrating additional diseases could lead to more comprehensive and holistic health surveillance, supporting decision-making across various health priorities.

While RCAS has been shown to possess several strengths, our findings also indicate areas that may need improvement. Identifying and addressing challenges related to data quality, user training, and potential barriers to system adoption are essential for optimizing the functionality and sustainability of RCAS. Additionally, exploring ways to scale the system’s capabilities for broader health applications will require careful planning and stakeholder engagement.

The positive outcomes and potential benefits of the implementation of RCAS extend beyond Rwanda’s borders. The lessons learned from the successes and challenges experienced with RCAS can inform the development and implementation of similar digital health systems in other countries that are facing public health emergencies. Additionally, our findings contribute to the growing body of evidence for the important role digital health can play in pandemic preparedness and responses.

## CONCLUSION

The findings from our assessment of RCAS indicate that the system efficiently provided data that were then readily available for decision-making related to COVID-19 case monitoring and vaccination coverage. The system’s key benefits, interoperability, and potential for expansion demonstrate its value in supporting ongoing public health efforts. However, addressing data quality issues and other challenges will be crucial for its sustained impact. The successful implementation of RCAS offers valuable insights for the global digital health community and paves the way for future advancements in disease surveillance and response.

## Data Availability

The data underlying this article are available in OSF repository, at https://osf.io/vs7ue/files/osfstorage/650d754ac0a3642df3189a2d.
